# *Heliotropium indicum* L.: From Farm to a Source of Bioactive Compounds with Therapeutic Activity

**DOI:** 10.1155/2021/9965481

**Published:** 2021-06-01

**Authors:** Chandan Sarkar, Milon Mondal, Bilkis Khanom, Md. Monir Hossain, Md. Solayman Hossain, Antoni Sureda, Muhammad Torequl Islam, Miquel Martorell, Manoj Kumar, Javad Sharifi-Rad, Ahmed Al-Harrasi, Ahmed Al-Rawahi

**Affiliations:** ^1^Department of Pharmacy, Bangabandhu Sheikh Mujibur Rahman Science and Technology University, Gopalganj 8100, Bangladesh; ^2^Department of Pharmacy, Mawlana Bhashani Science and Technology University, Tangail 1902, Bangladesh; ^3^Research Group in Community Nutrition and Oxidative Stress, University of Balearic Islands, Health Research Institute of Balearic Islands (IdISBa), and CIBEROBN (Physiopathology of Obesity and Nutrition), E-07122 Palma, Balearic Islands, Spain; ^4^Department of Nutrition and Dietetics, Faculty of Pharmacy, and Centre for Healthy Living, University of Concepción, Concepción, Chile; ^5^Universidad de Concepción, Unidad de Desarrollo Tecnológico, UDT, Concepción, Chile; ^6^Chemical and Biochemical Processing Division, ICAR–Central Institute for Research on Cotton Technology, Mumbai 400019, India; ^7^Phytochemistry Research Center, Shahid Beheshti University of Medical Sciences, Tehran, Iran; ^8^Facultad de Medicina, Universidad del Azuay, Cuenca, Ecuador; ^9^Natural and Medical Sciences Research Centre, University of Nizwa, Birkat Almouz 616, Oman

## Abstract

This study aimed to summarize the available data on the ethnomedicinal and phytopharmacological activities of *Heliotropium indicum* L. based on database reports. For this purpose, an up-to-date literature search was carried out in the Google Scholar, Scopus, Springer Link, Web of Science, ScienceDirect, ResearchGate, PubMed, Chem Spider, Elsevier, BioMed Central, and patent offices (e.g., USPTO, CIPO, NPI, Google patents, and Espacenet) for the published materials. The findings suggest that the plant contains many important phytochemicals, including pyrrolizidine alkaloids, indicine, echinitine, supinine, heleurine, heliotrine, lasiocarpine, acetyl indicine, indicinine, indicine *N*-oxide, cynoglossine, europine *N*-oxide, heleurine *N*-oxide, heliotridine *N*-oxide, heliotrine *N*-oxide, heliotrine, volatile oils, triterpenes, amines, and sterols. Scientific reports revealed that the herb showed antioxidant, analgesic, antimicrobial, anticancer, antituberculosis, antiplasmodial, anticataract, antifertility, wound healing, antiinflammatory, antinociceptive, antihyperglycemic, anthelmintic, diuretic, antitussive, antiglaucoma, antiallergic, and larvicidal activity. In conclusion, *in vitro* studies with animal models seem to show the potential beneficial effects of *H. indicum* against a wide variety of disorders and as a source of phytotherapeutic compounds. However, clinical studies are necessary to confirm the effects observed in animal models, determine the toxicity of the therapeutic dose and isolate the truly bioactive components.

## 1. Introduction

One of the barebones for the victory of principal health care is the accessibility and use of apposite drugs. Traditional medicine, since the early formation of human civilization, has been the most sensible and affordable source of treatment in the health care system, which is why people continue to rely on plants for multiple disorders [1]. The medicinal uses of each plant derive from the presence of significant amounts of various natural products, which can be used as alternative therapeutic or adjuvant tools. Medicinal plants play an energetic role in the discovery of new therapeutic agents, thus growing interest in the use of pharmaceutical consumption [2,3]. Medicinal plants contain many constituents such as alkaloids, flavonoids, tannins, phenols, saponins, and glycosides, with notable biological activities such as antimicrobial, analgesic, antipyretic, antitumor, wound healing, and cardioprotective, among others that can be useful against diverse human diseases [4,5].

**Figure 1:**
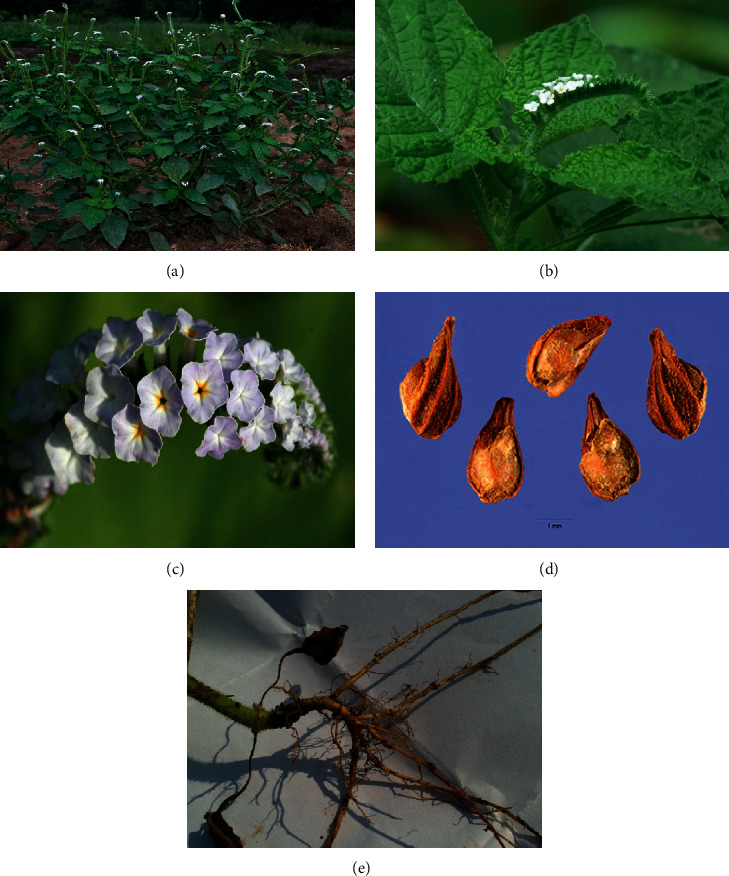
Different parts of *Heliotropium indicum* Linn:(a) whole plant, (b) leaves, (c) flowers, (d) seeds, and (e) roots.

*Heliotropium indicum* L. (family: Boraginaceae; Figure 1), locally known as “Hatisur” is derived from the Greek words “*helios*” meaning “sun” and “*tropein*” meaning “to turn,” indicating that the flowers and leaves turn toward the sun and known as the “Indian turnsole” [6]. It is also known as *Eliopia riparia* Raf., *Eliopia serrata* Raf., *Heliophytum indicum* (L.) DC., Heli*otropium africanum* Schumach. & Thonn., *Heliotropium cordifolium* Moench, *Heliotropium foetidum* Salisb., *Heliotropium horminifolium* Mill., and *Tiaridium indicum* (L.) Lehm. *H. indicum* is distributed throughout Bangladesh, Nepal, Sri Lanka, Thailand, India, and other areas of tropical Asia and in some parts of Africa [7]. *H. indicum* is a small annual or perennial herb with a height of about 15–50 cm in length, with the leaves always opposite, and the stem and root covered by a hairy layer [7]. Flowering time is around the whole year, and flowers are calyx green; the fruits are dried and consist of 2–4 free or almost free nutlets in 4–5 mm long [8].

Traditionally, this plant is widely used against many pathological disorders including wound healing, antidote, bone fracture, febrifuge, cures eye infection, menstrual disorder, nerve disorder, kidney problem, and antiseptic purpose [9–14]. *H. indicum* contains many important phytochemicals such as tannins, saponins, steroids, oils, and glycosides [12,15]. Schoental [16] and Hartmann and Ober [17] isolated pyrrolizidine alkaloids (e.g., indicine *N*-oxide, heliotrine, etc.) from this plant. Scientific reports suggest that *H. indicum* possesses many important pharmacological activities, including antiinflammatory [18], wound-healing [19], anticancer [15], and anticataract activities [20].

This review aims to show the current scenario on the ethnomedicinal, phytochemical, and pharmacological profiles of *H. indicum*.

## 2. Plant Taxonomy

The taxonomic hierarchy of *H. indicum* is the following:

  Domain: Eukaryota
  Kingdom: Plantae
  Phylum: Spermatophyta
  Subphylum: Angiospermae
  Class: Dicotyledonae
  Order: Boraginales
  Family: Boraginaceae
  Genus: *Heliotropium*
  Species: *Heliotropium indicum* L

## 3. Plant Morphology

*H. indicum* is an erect, thick fetid, annual or perennial herb with hirsute ascending branches, reaching between 20 and 60 cm in height [13]. The leaves are opposite or sub-opposite, alternate or sub-alternate and straight forward, sheet-shaped from ovate to elliptical, hairy, and sharp and 5–10 cm long. The margins of the leaves are undulate; the nerves present on both sides are serrulate or cordate and clearly visible under the leaves [21]. The petiole is about 1–7 cm long, while the flowers progress apically within the cymose; at maturity, nutlets are present at the base of the inflorescence. Generally, flowers are white or whitish violet in color, regular, sessile, axillary, and nearly 5 mm in diameter. Sepals are diffused with hairs outside, deep green in color, linear to lanceolate, uneven or unequal, and about 5–3 mm long. The fruits are dry and 2–4 lobed, with or without united nutlets, and 3–6 mm long. This species grows in sunny places preferring heights around 800 m [22]. Botanical descriptions of *H. indicum* are given in Table 1.

## 4. Methodology

The literature search was performed using the databases: Google Scholar, Scopus, SpringerLink, Web of Science, ScienceDirect, ResearchGate, PubMed, ChemSpider, Elsevier, BioMed Central, and USPTO, CIPO, INPI, Google Patents, and Espacenet. The scientific databases were chosen based on the topic covered (i.e., ethnobotany, ethnomedicinal uses, ethnopharmacology, pharmacology, phytochemistry, and therapeutic value) and geographical coverage (i.e., Asia and Africa). The common keyword “*Heliotropium indicum*” was used to search published materials, which was then paired with “traditional uses,” “ethnopharmacology,” “phytochemistry,” “pharmacology,” and “toxicity.” Other literature sources included papers published in international journals; reports from international, regional, and national organizations; conference papers; and related books. Chemical structures were drawn using the software ChemSketch (Version 14.01).

## 5. Traditional and Folk Values

Ethnopharmacology is the study of medicinal plant use in specific cultural groups or the study of differences in response to drugs in different cultures [23]. About 90% of native people depend on the natural products of plant origin to treat several diseases [24]. With the knowledge of ethnopharmacology, the whole plant of *H. indicum* has been traditionally used in different folklore systems to cure several diseases in different countries over the world. In Bangladesh, the juice or decoction of leaves and roots of *H. indicum* is traditionally used in chicken pox, allergy, blood purification, swelling of the knees, joint pain, and severe itchy legs and also be used as an antidote to poisoning [12,25–27]. In India, different parts of the herb, mainly leaves as a paste or beverage, are used on wounds, skin infections, ophthalmia, snakebite, and scorpion sting [28,29]. The decoction of both root and leaf is used to handle whooping cough in children in eastern Nicaragua [13].

The infusion of flowers at low doses is applied to regulate the menstrual cycle, while large doses for abortion by introducing into the vaginal cavity. In Jamaica, the flower infusion is used by females to treat menorrhagia, while in Senegal and the Philippines, it is used to treat kidney stone [9,10]. In the Philippines, the decoction of dried roots is drunk to encourage menses, while the seeds are used to heal wounds and treat cholera and malaria [30]. In African countries, it is reported that this plant is useful in treating malaria, dermatitis, abdominal pain, renal failure, and urinary infections [9,31,32]. In Thailand, the dried and powdered inflorescence (1 gm) mixed with milk or water is used for three days beginning with the fourth day of menses to yield permanent sterilization in females [33]. The whole plant is used to treat ringworm infection and counteract putrefaction in Malaysia, while the decoction of the whole plant is applied to treat gonorrhea in Burma [30]. The leaf juice is used to treat the stings and boils of scorpions and insect bites. On the other hand, the boiled juice with castor oil is used to treat mad dog bite infections [34]. Moreover, *H. indicum* is also used to treat rheumatism [35], ulcer, venereal disease, fever, sore throat, and sores in the rectum [36]. Traditional uses of *H. indicum* in different countries are summarized in Table 2.

## 6. Phytochemical Constituents

**Figure 2:**
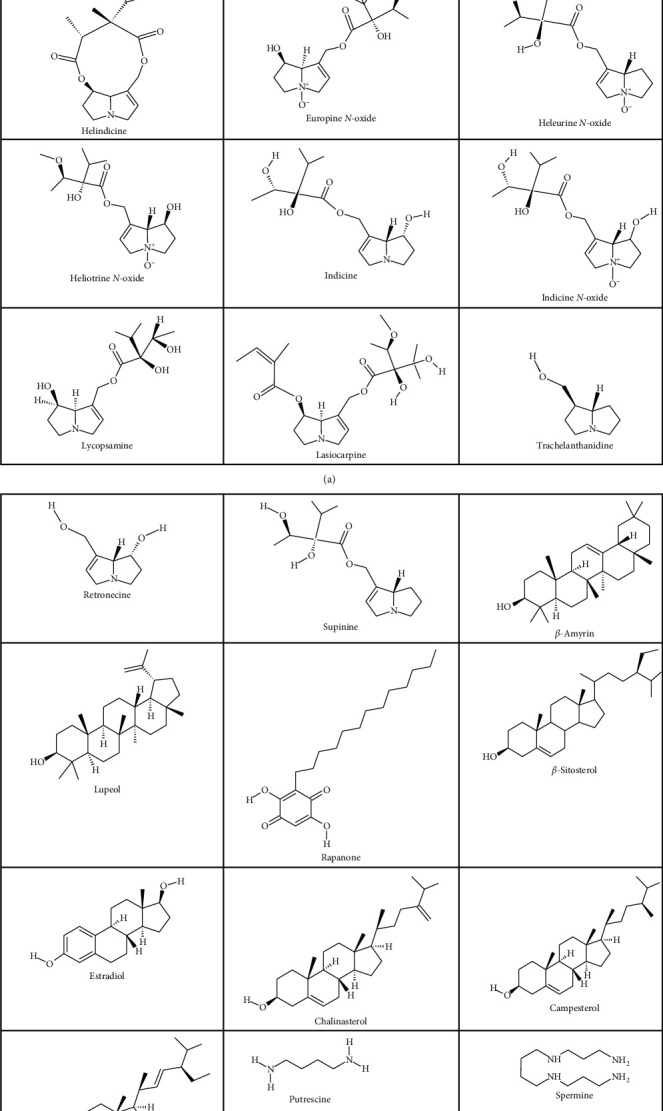
Some important isolated compounds from *H. indicum* L.

Based on the history of traditional and folk medicinal uses of *H. indicum*, many researchers have been investigating its phytochemical and pharmacological properties to identify the compounds responsible for its wide use as herbal medicines. The plant contains many important phytocomponents, including alkaloids (e.g., acetyl indicine, cynoglossine, echinitine, heleurine, heliotrine, helindicine, europine *N*-oxide, heleurine *N*-oxide, heliotridine *N*-oxide, heliotrine *N*-oxide, indicine, indicinine, indicine *N*-oxide, lasiocarpine, lycopsamine, trachelanthamidine, retronecine, and supinine), triterpenes (e.g., *β*-amyrin, lupeol, rapone, and rapanone), sterols (e.g., *β*-sitosterol, estradiol, chalinasterol, campesterol, hexacosane-1-ol, and stigmasterol), amines (e.g., putrescine, spermidine, and spermine), and volatile oils (e.g., 1-dodecanol, *β*-linalool, and phytol) [30,53,62,65,67–72]. Two new alkaloids, namely, heliotrine and indicine *N*-oxide, along with other alkaloids, including heleurine, supinine, echinitine, heliotrine, lasiocarpine *N*-oxide, acetyl indicine, indicinine, and retronecine, have been isolated from the aerial parts of *H. indicum* [68, 71, 73, 74]. Europine *N*-oxide, cynoglossine, heliotrine *N*-oxide, heleurine *N*-oxide, and heliotridine *N*-oxide were isolated from the seeds of this plant. Another new pyrrolizidine alkaloid, helindicine, has also been isolated from the roots of *H. indicum* [75]. The reported compounds are presented in Table 3, and the main representative compounds are shown in Figure 2.

## 7. Pharmacological Activities

Various solvent extracts (e.g., aqueous, chloroform, ethanolic, methanolic, and petroleum ether) of the whole plant of *H. indicum* as well as its various parts (e.g., root, stem, leaf, etc.) have been investigated to validate the folk value, and the results showed diverse biological effects on experimental animals, which are described in the present section. Pharmacological activities of different parts of *H. indicum* have been shown in Table 4.

### 7.1. Antioxidant Activity

The methanolic extract of various parts of the plant, such as leaf, stem, and roots, was used to measure the total phenolic compounds and flavonoids contents as well as to determine DPPH free radical scavenging activities. The inflorescence extracts presented a higher concentration of total phenolics and flavonoids with a 21.70 mg gallic acid equivalent per gram (GAE/g) and 4.90 mg quercetin equivalent per gram (QE/g), followed by leaves, stems, and roots. The percentage of free radical scavenging activity of the methanolic extracts of inflorescence, leaves, stems, and roots followed the same response pattern, with the maximum values for inflorescence (77.78%) followed by leaves (55.25%), stems (47.49%), and roots (<20%) with respect to the standard gallic acid and ascorbic acid [80]. In another study by the same authors, the potential antioxidant activity of methanolic extracts of callus of *H. indicum* cultured for 30days at different temperatures (20, 25, 30, and 32°C) reported the highest DPPH scavenging activity (IC_50_ = 53.17 ± 1.43 *μ*g/mL) at 30°C respect to the other temperatures [81]. In addition, another study reported that the ethanolic extract of *H. indicum* exerted more antioxidant capacity (EC_50_: 28.91 ± 4.26 *μ*g/mL) than the water extract (EC_50_: >100 *μ*g/mL) [14].

### 7.2. Analgesic Activity

The analgesic effect of the ethanolic and aqueous extracts of the aerial parts of *H. indicum* (30–300 mg/kg) in a mouse model of formalin-induced pain was compared with the standard drugs, diclofenac sodium (1–10 mg/kg), and morphine (1–10 mg/kg). The neurogenic and inflammatory phases of the formalin-induced nociception were inhibited dose-dependently by both the aqueous and ethanolic extracts, suggesting a potential analgesic application [82,101]. However, toxicity studies reported that 14-day oral administration of 1–2 g/kg of *H. indicum* aqueous extracts induced pathologic effects on the heart, kidney, liver, and lungs; therefore, prolonged and continuous use is not recommended.

### 7.3. Antinociceptive Activity

The methanol root extract of *H. indicum* exhibited 34.76 and 64.67% writhing inhibition in Swiss albino mice at 250 and 500 mg/kg of body weight (po), respectively, whereas the standard drug diclofenac sodium showed 66.67% writhing inhibition at the clinically established dose of 25 mg/kg for mice [7]. Another study suggested that the chloroform extract of leaves of *H. indicum* showed maximum antinociception effect (82.79%) at 150 mg/kg of body weight in the hot-plate test in male Swiss albino mice that was compared with the standard drug, pentazocine [83].

### 7.4. AntiInflammatory Activity

The antiinflammatory activity of methanolic root extracts of *H. indicum* (100 mg/kg) was assayed against carrageenin-induced acute paw edema and cotton pellet granuloma sub-acute inflammation models, and the standard drugs acetylsalicylic acid for the acute assay and phenylbutazone for the sub-acute assay were used as positive controls [18]. The extract evidenced a significant antiinflammatory activity with a 49.05% reduction in paw edema and 55.09% reduction in granuloma formation. These results were similar to those obtained by positive controls using the same concentration of 100 mg/kg. In another study, the ethanolic and petroleum ether extracts of *H. indicum* (25 mg/kg) were investigated in an egg-white-induced acute paw edema rat model [84]. Both extracts evidenced notable antiinflammatory effects, reporting similar values to the standard reference ketorolac trimethamine (10 mg/kg). The chloroform leaf extract of *H. indicum* extract (150 mg/kg of body weight) also showed a significant antiinflammatory effect (80.0%) on carrageenan-induced paw edema in albino Wistar rats [83]. An aqueous whole plant extract of *H. indicum* (30–300 mg/kg, p.o.) showed an antiinflammatory effect on the lipopolysaccharide-induced uveitic rabbits. The extract and prednisolone (positive control) expressively reduced both the clinical scores of inflammation and inflammatory cell infiltration compared with the negative control group [85]. A pharmaceutical oral product obtained from *H. indicum* is used against acute and chronic inflammation, particularly against inflammatory diseases of the intestines [102].

### 7.5. Antimicrobial Activity

The alcoholic extract with a percentage yield of 7.2% w/w of the whole plant showed a concentration-dependent (1–100 mg/mL) antibacterial activity against *Bacillus subtilis, Bacillus pumilus, Staphylococcus aureus, Micrococcus glutamicus, Pseudomonas aeruginosa, Proteus vulgaris, Serratia marcescens*, and *Escherichia coli*. The alcoholic extract also showed antifungal activity against *Aspergillus niger, Aspergillus wentii, Rhizopus oryzae, Saccharomyces cerevisiae*, and *Candida albicans* [8,86]. However, as high extract concentrations are required to observe inhibitory effects, activity-directed assays are necessary to isolate and characterize the active metabolite responsible for the observed activity. The petroleum ether, chloroform, aqueous, and methanolic extracts of *H. indicum* leaves showed antimicrobial activity against both Gram-positive and Gram-negative bacteria, such as *B. subtilis, S. aureus, P. aeruginosa*, and *E. coli* [6,37]. In a wound infection model with *S. aureus* and *P. aeruginosa*, the methanolic and aqueous extracts of leaves mixed with a simple ointment (10% w/w) presented the most promising activity favoring the healing similarly to the reference standard nitrofurazone [37]. In another study, the antimicrobial screening of petroleum and methanolic extracts of the aerial parts of the plant evidenced significant zones of inhibition against the three previously mentioned microorganisms [6].

The aqueous ethanol and chloroform extract of the whole plant of *H. indicum* showed antibacterial and antifungal activities, where it produced significant zones of inhibition against 70% of the tested organisms, using amikacin (5 g/disc) as a positive control [87]. Among the different extracts, chloroform is the one that showed the best results, although the zone of inhibition was always lower than for amikacin (e.g., for *S. aureus*, the inhibition diameter was 19 mm for the control and 12 mm for chloroform extract). The methanol extract of the whole plant also showed activity against five Gram-positive and eight Gram-negative bacteria and three fungi, using the standard antibiotic, ciprofloxacin, as a positive control [88]. In addition, the carbon tetrachloride soluble materials obtained by the fractionation of the methanolic extract using a rotary evaporator revealed notable activity against a number of microbes with zones of inhibition ranging from 7 to 20 mm, showing the highest inhibitory capacity for *Bacillus cereus* (20.0 mm) [88]. The methanol extract of *H. indicum* leaves (6.25, 12.5, 25, 50, 100, and 200 mg/mL) showed activity against *S. aureus*, *P. aeruginosa, Proteus mirabilis*, and *E. coli*, where the diameters of the zones of inhibition were 6 mm [8]. However, the high concentration required to obtain inhibition, compared with the positive control (gentamycin, 10 mg/ml), suggests a low antimicrobial capacity of the extract. The volatile oil isolated from the aerial parts of *H. indicum* with phytol (49.1%), 1-dodecanol (6.4%), and *β*-linalool (3.0%) as main compounds showed antituberculosis activity against *Mycobacterium tuberculosis* H37Ra with an MIC value of 20.8 *μ*g/mL, using the drugs, isoniazid, and kanamycin, as positive controls [79].

### 7.6. Antihyperglycemic Effect

Administration of the whole plant methanol extract among the different solvent extracts of *H. indicum* (250, 500, 750, or 1,000 mg/kg) on the fasting blood glucose levels of streptozotocin-induced (STZ-induced) diabetic rats showed a significant reduction (31.5%) but less antihyperglycemic activity in comparison with the aqueous extract (47%) and methanol active fraction (750 mg/kg of body weight) of the plant (60%) [89].

### 7.7. Anticataract Effect

The ethanolic leaf extract of *H. indicum* (200 mg/kg of body weight) showed a significant anticataract activity in rats. The results showed that there was a significant increase in the lens glutathione, soluble protein, and water content in the groups of *H. indicum* and vitamin-E-treated animals than the galactose-containing control group [20]. Another study showed that the aqueous extracts of the whole plant (including aerial and root parts) significantly inhibited the development of selenite-induced cataracts in Sprague–Dawley rats [90].

### 7.8. Antiplasmodial Properties

In order to find out its scientific relevance to the traditional use in malaria, the extracts of *H. indicum* were undergone for the evaluation of antiplasmodial activity. However, *H. indicum* methanolic extracts had not shown clear antiplasmodial effects assayed *in vitro* against chloroquine-resistant (K1) and sensitive (FCR3) strains, and anti*Trypanosoma* effects were assayed in *Trypanosoma brucei brucei* GUT at 3.1 strain [91]. Its use in traditional medicine can be explained by its activity in reducing hyperthermia and colic, which are two symptoms of malaria [103].

### 7.9. Antifertility Activity

Antifertility and abortifacient activity of petroleum ether extract of *H. indicum* were significant in rats, which validated the ethnomedicinal use of this plant as an antifertility agent [77]. The n-hexane and benzene fractions of the ethanol extract of the whole plant also showed antifertility activity using antiimplantation and abortifacient models in rats [92].

### 7.10. Anthelmintic Effect

The anthelmintic effects of methanolic and aqueous leaf extracts of *H. indicum* (25, 50, and 100 mg/mL) were tested against the Indian adult earthworm, *Pheretima posthuma*. Mebendazole was used as a reference standard using the same concentrations as the extract. The time to paralysis and death progressively decreased in parallel with the increase in the concentrations of the methanolic extract, showing results similar to those of the standard drug mebendazole [93]. On the contrary, the effects of the aqueous extract were much smaller and not very effective against *P. posthuma*.

### 7.11. Anticancer Effect

The methanolic extract of *H. indicum* roots (10, 20, 40, 80, and 160 *μ*g/mL) showed a potent cytotoxic effect on the brine shrimp nauplii [7]. The LC_50_ values were ranged from 2.57 to 31.44 *μ*g/mL. The crude methanol extract also showed cytotoxic effects on brine shrimp nauplii with the LC_50_ value of 2.57 ± 0.22 *μ*g/mL as compared with 0.45 *μ*g/mL for positive control vincristine sulphate [88]. In another study, the anticancer effects of the methanolic extracts of stem and leaves were investigated against HeLa cell line [94]. Both methanolic extracts exhibited antiproliferative activity after 48 h of treatment, evidencing a relative death percentage of 64.5% for the methanolic extract of stem at 200 *μ*g/mL and 49.7% for the leaf extract at the same concentration with respect to control cell supplemented only with the vehicle [94]. The ethanolic extract of the whole plant was also found to exert a significant antiproliferative effect on SKBR3 human breast adenocarcinoma cell line [91]. Indicine *N*-oxide, which is the principal pyrrolizidine alkaloid isolated from this plant has reached phase 1 clinical trial in advanced cancer patients with the risk of hepatotoxicity [104].

### 7.12. Antitussive Effect

The ethanolic leaf extract of *H. indicum* showed an antitussive effect on experimental animals. While statistically comparable with dextromethorphan, the results of the investigation showed that 50% and 100% ethanolic extract syrup reduced the coughing score by 4.67 and 2.0, respectively [95].

### 7.13. Antiglaucoma Activity

The aqueous whole plant extract of *H. indicum* (30–300 mg/kg of body weight) significantly reduced the intraocular pressure in acute and chronic glaucoma, preserved glutathione levels, and glutamate concentration in rabbits [90].

### 7.14. Wound Healing Capacity

The alcoholic extract of *H. indicum* showed wound-healing activity in animal models. In a rat model, topical application of 10% w/v *H. indicum* showed a complete wound-healing capacity on the 14^th^ day [19]. Two alkaloids, pestalamide B and glycinamide, *N*-(1-oxooctadecyl) glycyl-lalanylglycyl-L-histidyl, isolated from the *n*-butanol crude extract of *H. indicum* showed excellent wound-healing activity on H292 human lung cells [96]. The *n*-butanol extract of *H. indicum* also showed a significant wound-healing activity on H292 human lung cells *in vitro* [96]. Another experiment proved that the methanol and aqueous extracts of *H. indicum* revealed significant wound-healing activities than the other extracts (e.g., petroleum ether and chloroform) in rats [37].

### 7.15. Gastroprotective Effect

The aqueous extract of the dried leaves of *H. indicum* showed a dose-dependent gastroprotective effect in indomethacin-induced (80 mg/kg of body weight) gastric ulcer mucosa in rats [10]. Histological observations of the different components of the mucosa layer of the stomach evidenced normal morphological appearance in the *H. indicum* groups, whereas in the control group, significant erosions in the mucosa were observed. It was also supposed that this effect may be due to the presence of tannins, alkaloids, and saponins in the leaves of the plant that may induce the release of prostaglandins in gastric mucosa maintaining gastric microcirculation through mucus and bicarbonate production.

### 7.16. Diuretic Effect

The methanolic root extract of *H. indicum* at 200 and 400 mg/kg revealed a marked diuretic effect of the electrolyte loss ratio (Na^+^/K^+^ excretion ratio was 1.38 and 1.45, respectively) as compared with the standard diuretic furosemide (1.37) in mice [7,105].

### 7.17. Relaxant/Receptor Property

The ethanol (95%) extract of the roots showed weak smooth muscle-relaxant activity on guinea pig ileum and rabbit duodenum [97]. Another study performed on isolated guinea pig ileum, rabbit jejunum, rat uterus, and rat anococcygeus preparations with several agonists, antagonists, and the aqueous plant extract showed a dose-dependent activity of the acetylcholine, methylcholine, carbamylcholine, nicotine, histamine, oxytocin, and plasma cholinesterase [98].

### 7.18. Antithrombotic Effects

Different extracts of *H. indicum* exhibited a potential lysis of clots and stabilizing activities of the membrane, which is why traditionally the leaves of *H. indicum* have been used as a remedy for thrombosis. The ethanol, petroleum ether, carbon tetrachloride, and chloroform extracts of *H. indicum* leaves showed 23.78, 35.40, 32.48, and 18.95% clot lysis activity, respectively, in the blood of healthy male subjects [99]. In this study, streptokinase, used as a positive control, showed a 65.15% clot lysis activity. In another study, the methanolic extract of the whole plant showed mild-to-moderate thrombolytic activity at a concentration of 1.0 mg/mL protecting red blood cells against hypotonic and heat-induced hemolysis [88]. In addition, the carbon tetrachloride soluble fraction obtained from this extract showed a 41.47 ± 1.12 and 37.97 ± 0.14% of red blood cell lysis induced by hypotonic solution and heat, respectively, while acetylsalicylic acid used as positive control showed 71.92 and 42.12% of lysis [88].

### 7.19. Larvicidal Activity

*H. indicum* is a potential plant for the control of *Aedes aegypti*, which is a potential vector of the dengue virus. Veerakumar et al. [106] suggested that *H. indicum* can be an ideal eco-friendly plant for the control of *Anopheles stephensi* and *A. aegypti*. The alcoholic extracts of *H. indicum* at different concentrations (0.30, 0.25, 0.20, 0.15, 0.10, 0.075, 0.050, and 0.025 mg/mL) were found to act against the mosquito larvae of *A. aegypti* [100]. In this study, an inability to come to the surface, restlessness, loss of equilibrium, and finally the death of the larvae were observed with the treatment of *H. indicum* extracts. The results showed a mortality of 10% already in the lowest concentration of 0.025 mg/mL, reaching 100% in the concentration of 0.25 mg/mL. However, no positive control was used in the study, making it difficult to compare the real efficacy of the extract.

### 7.20. Miscellaneous Effects

The aqueous and ethanol extracts of the *H. indicum* roots exhibited a strong uterine stimulant effect in rats [107]. Bero et al. [103] reported that the aqueous extract of *H. indicum* possesses antileukemic and ganglion-blocking activities. The leaf extract of *H. indicum* is also evident to be used in ophthalmic disorders, erysipelas, and pharyngodynia [108]. An aqueous whole plant extract of *H. indicum* (30–300 mg/kg of body weight) exhibited an antiallergic effect on Dunkin–Hartley guinea pigs possibly by immunomodulation pathway [90].

### 7.21. Toxicological Profile

The aqueous and ethanolic extracts of the whole plant exhibited cumulative toxic effects on the kidney, liver, and lungs on prolonged use [82,101]. Heliotrine is evident to cause liver damage in experimental animals [109], while lasiocarpine developed malignant tumors in rats [110]. Retrorsine exerted a toxic effect on human embryo liver cells [111].

In a five-month toxicity study, an oral administration of the ethanol extract of *H. indicum* caused dose-dependent mortality (LD_50_: 9.78 g/kg of body weight) in Swiss albino mice [112].

Pyrrolizidine alkaloids are evident to produce highly reactive adducts, such as 2,3-dihydro-1H-pyrrolizine protein, through the hepatic cytochrome P450 system. These adducts bind to proteins and genetic materials (e.g., DNA and RNA) and induce veno-occlusive disease in the liver [113]. The acute intoxication of pyrrolizidine alkaloids is characterized by hemorrhagic necrosis, hepatomegaly, and ascites, while chronic exposure is characterized by necrosis, fibrosis, cirrhosis, liver failure, and even death [114]. Due to photosensitization in animals upon their consumption and metabolism, pyrrolizidine alkaloids may initiate skin cancer [115]. Moreover, these substances can cause neurotoxicity and encephalitis, which is characterized by vertigo, headaches, delirium, and loss of consciousness [116].

## 8. Discussion

*H. indicum* has long been used in traditional medicine systems to treat various ailments; therefore, this review summarized the botany, traditional uses, phytochemistry, and pharmacology of this plant and its components. A number of phytochemical classes have been isolated from this medicinal plant. Available pharmacological studies on the ingredients and crude extracts indicated broad biological effects of *H. indicum*, providing basic evidence for traditional claims. However, as viewed from the current findings, some areas still require scientific evaluation and exploration. First, the leaves of *H. indicum* are the main medicinal part used in Bangladesh, while in other countries (e.g., India and Thailand), different parts are used for different purposes. Therefore, it is convenient to investigate the differences between plant parts regarding phytochemistry and pharmacology. Second, alkaloids are considered as the main bioactive constituents, particularly heliotrine and heleurine *N*-oxide. Numerous bioactivities of other bioactive constituents have been reported to be of prominent pharmacological activities and are worth to be given more attention. In addition, more research on the identification and isolation can be done on extracts, with reported bioactivities to discover new active phytochemicals and elucidate their structure-activity relationships and possible synergistic effects. Third, the reliability of the herb to treat coronary heart disease, kidney diseases, hemorrhagic diseases, and vitiligo has been justified by the long history, but current findings are not enough to ascertain these traditional claims from the perspective of modern pharmacology. Moreover, the evaluation of representative and appropriate cell or animal models is equally important to assess these traditional uses precisely. Fourth, the anticancer activity of *H. indicum* indicated that the plant could be a natural source to find promising and cost-effective lead compounds with little side effects for cancer treatment. The cytotoxic effects are mainly due to the action of the pyrrolizidine alkaloid, indicine *N*-oxide, which alters the assembly of tubulin into microtubules, inducing DNA damage [117]. However, the appearance of liver toxicity and even bone marrow aplasia has led to the withdrawal of this compound from the development of clinical trials [118,119]. Thus, it will be necessary to find new compounds in *H. indicum* with anticancer potential. Finally, acute and chronic toxicity should be comprehensively studied in order to establish safety and toxicological limits and provide guidance for clinical applications.

Phytochemical research has led to the isolation and identification of 32 compounds in *H. indicum* [13, 22]. Different classes of compounds have been detected, including alkaloids, triterpenes, sterols, amines, and volatile oils (Table 3 and Figure 2). *H. indicum* contains a large class of alkaloids with antiinflammatory, analgesic, antibacterial, antitumor, and other activities. Among them, acetyl indicine, echinitine, heleurine, heliotrine, indicine, indicinine, indicine *N*-oxide, lasiocarpine, retronecine, supinine, and trachelanthamidine were isolated from the aerial parts of the plant, while cynoglossine, europine *N*-oxide, heleurine *N*-oxide, and heliotridine *N*-oxide were separated from the seed, and heliotrine and lycopsamine were separated from the root [30, 53, 62, 67, 69, 71, 73, 75, 76]. The chemical structures of alkaloids are shown in Figure 2. Indicine *N*-oxide, which is the principal pyrrolizidine alkaloid isolated from *H. indicum*, has the potential risk of hepatotoxicity [104], and because of the presence of a high amount of pyrrolizidine alkaloids, this plant exerts potent anticancer activity [94]. The plasma cholinesterase receptor activity of *H. indicum* validates some of its traditional folk values such as relieving abdominal pain, hypertension, and impotence and sexual weakness [98].

Triterpenes are the second class of molecules that have been well-studied in *H. indicum* evidencing a wide variety of biological functions. Among them, *β*-amyrin, lupeol, and rapanone have been evidenced to possess biological functions, including defense against herbivores, microbial attack, or other sources of injury [71, 77]. *β*-Amyrin also showed potential antihyperglycemic and hypolipidemic effects, suggesting that it could be a lead compound for drug development for diabetes and atherosclerosis [120]. Lupeol is a novel antiinflammatory and anticancer dietary triterpene, which has strong antioxidant, antimutagenic, antiinflammatory, and antiarthritic characteristics with potential pharmaceutical applications [121]. Rapanone has been reported to exert significant antioxidant, antiinflammatory, and cytotoxic activities against a panel of human tumor cells [122]. Toxicity studies have observed some alterations in rats such as tremor, ataxia, increased respiratory rate, and decreased activity at concentrations of *β*-amyrin above 30 mg/kg for 4 weeks, while no toxicity has been observed for lupeol at doses up to 200 mg/kg [121, 123]. Although no significant effects of rapanone have been shown in non-cancer cells, at doses of 60 and 120 mg/kg, it induced anovulatory effects in female mice [124, 125].

Six main sterol compounds have been isolated from *H. indicum*: *β*-sitosterol, chalinasterol, campesterol, stigmasterol, hexacosane-1-ol, and estradiol [77, 78]. Sterols have a wide variety of functions in plant physiology, including the regulation of Na^+^/K^+^-ATPase, cell differentiation, and proliferation or membrane fluidity and permeability [126–128]. In addition, plant-derived sterols have been reported to exert antiinflammatory effects useful in the treatment of non-alcoholic fatty liver, inflammatory bowel diseases, and allergic asthma [129]. However, no studies have specifically evaluated the effects of sterols isolated from *H. indicum* against these diseases.

Amines are an important class of molecules in *H. indicum* that display pesticidal, fungicidal, herbicidal, analgesic, and antioxidant activities. Putrescine, spermidine, and spermine were separated from the leaves of *H. indicum* [76]. Putrescine scavenges reactive oxygen species and regulates DNA and protein synthesis, cell proliferation, and differentiation of tissues, thereby supporting placental development and embryogenesis in mammals [130]. Spermidine is a polyamine compound that counteracts aging and promotes cellular longevity [131]. The compound induces autophagy in a mammalian target of rapamycin (mTOR) independent manner by inhibiting the acetyltransferase EP300, resulting in hypoacetylation of several core autophagy proteins, including ATG5, ATG7, ATG12, and LC3 [132]. Spermine is a natural polyamine known to be essential regulators of various cellular processes, including DNA stability, cellular growth, differentiation, and apoptosis, and also used to treat cancer, other pathologies, inflammation, immunity, infection, and aging [133].

Three volatile oils were separated from the whole plant of *H. indicum* [79]. Among them, linalool (acyclic monoterpene alcohol) exerted its antiproliferative activity against various cancer cells through the mevalonate pathway [134]. Linalool has nutraceutical anticancer, antioxidant, antimicrobial, antidiabetic, antinociceptive, antiinflammatory, and hypolipidemic effects [135]. Phytol, diterpene alcohol, inhibits the inflammatory response by reducing cytokine production and oxidative stress and also provides antinociceptive activities [136, 137], and it has many biomedical applications [138, 139], including antimicrobial, cytotoxic, anticancer, non-mutagenic, antiteratogenic, antibioticchemotherapeutic, antidiabetic, lipid-lowering, antispasmodic, anticonvulsant, antinociceptive, antioxidant, antiinflammatory, anxiolytic, antidepressant, immune-adjuvant, hair growth facilitator, hair fall defense, and antidandruff activities [140]. Moreover, it has antipyretic [141] and clot lysis activities [142].

Diabetes mellitus is a chronic metabolic disease caused by an absolute or relative lack of insulin and/or reduced physiological insulin activity, resulted in hyperglycemia and abnormalities in carbohydrate, protein, and fat metabolism [143]. The methanol extract of *H. indicum* showed a dose-dependent antidiabetic effect on STZ-induced diabetic rats [89]. Different compounds present in this herb, for example, lupeol [144], phytol [140], and stigmasterol [145], have been found to show antidiabetic effects in experimental animals.

Rapanone has cytotoxic effects on MCF-7 cells, where it induced apoptosis through mitochondrial membrane potential loss [146]. Although effective therapeutic interventions are yet to be found out, it has been seen that estradiol positively impacts some aspects of cognitive function in humans and other animals [147]. Stigmasterol also possesses many biological activities, including immune-modulatory [148], anticancer [149], neuroprotective [150], hypolipidemic [151], and other effects. Putrescine has antiaging property [152] and can reduce antibiotic-induced oxidative stress in *Burkholderia cenocepacia* [153]. Spermidine alleviated autoimmune encephalomyelitis by inducing inhibitory macrophages [154]. It has several important biological activities, including antioxidant [155], cardioprotective, [156], neuroprotective [157], and other effects.

Spermine is a polyamine, initially discovered as crystals in human semen by Antonie van Leeuwenhoek in 1678 [158], and evokes olfactory responses in teleost fish [159] and possibly humans [160]. In a study, it has been found to act as a specific semen-derived sex pheromone in sea lamprey and promotes mating behaviors [161]. It is also evident to show antiinflammatory [162], mitochondrial protein synthesis [163], cardioprotective [164], and other effects. Lycopsamine exerted protective effects in spinal cord injury in rats by improving functional recovery and suppressing apoptosis [165]. Lupeol, a triterpene, found in this plant has several bioactivities, including antidiabetic, antiinflammatory, antioxidant [166, 167], skin protective [168], anticancer [149], and so on.

## 9. Conclusion and Future Directions

Medicinal plants and traditional medicine comprise about 90% of newly discovered pharmaceuticals, thus ensuring the safety, quality, and effectiveness of medicinal plants and herbal drugs that have gained much attention nowadays [169, 170]. Numerous results of experiments developed by researchers around the world support the biological activities associated with the traditional uses of *H. indicum*. In this sense, it can be concluded that *H. indicum* is a potential source of chemical compounds with promising biological activities. However, nowadays, clinical trials are scarce, which makes it difficult to translate them into routine clinical practice, making it necessary to carry out additional studies. In addition, several pyrrolizidine alkaloids isolated from the plant have been evident to show hepatotoxic effects on experimental animals; hence, further studies are required to ensure the safety of internal use of this plant. We hope that the information provided here could be helpful for the safe traditional uses and beneficial for further research.

The use of plant extracts in experimentation involves many drawbacks, including changes in their constituents depending on the climate or form of cultivation, presence of compounds with adverse or antagonistic effects, or changes in bioactivity during their handling, storage, or preparation of materials. Thus, working with pure compounds with known bioactivity makes it possible to obtain a targeted therapeutic effect and determine effective doses, toxic doses, and selectivity indexes to control the quality of the therapeutic formulation [171]. In addition, working with isolated compounds will reduce the risk of infections in the plant that could end up affecting patients and the presence of contaminants such as heavy metals [172].

Loss of medicinal plant species over time is another challenge for us. Among 80,000 flowering plant species that are used for pharmaceutical purposes, about 15,000 species are exposed to a risk of extinction due to high harvesting and destruction of habitats [173], and 20% of their wildlife resources are decreasing due to growing human populations and excessive consumption of plants [174]. Thus, the environmental code of ethics should be strictly followed to preserve the biodiversity of medicinal plants [175]. The good agricultural practice may be helpful for the production and quality assurance of medicinal plants [176]. For example, China has promoted the growth of conventional medicinal plants [177].

Nowadays, many people believe that using herbal medicines is good for health, but there are still many concerns about its safety and efficacy. The ethnobotanical record of *H. indicum* indicates that this plant is used in many countries around the world for various diseases. Upon going through the scientific reports on this plant, it should be claimed that *H. indicum* contains many important phytochemicals and possesses diverse biological activities, suggesting it as an important medicinal plant. More studies are necessary on its phytochemical analysis. Furthermore, the biological activities evaluated on its phytoconstituents are not sufficient. Although *H. indicum* can potentially contribute to the advancement of health care, to date, only a few studies have been conducted on its isolated constituents, limiting its translation to clinical practice. Another factor that hinders its clinical use is the presence of some components, such as heliotrine, lasiocarpine, and retrorsine, with evidence of toxic effects on experimental animals or human-derived cells. In addition, to build credibility for the use of this medicinal plant in conventional medicine, the empirical arguments should be converted into evidence-based arguments. Finally, several issues about safety, effective dosing, treatment duration, side or adverse effects, acute and chronic toxicities, as well as the standardization of *H. indicum* herbal preparations and phytoconstituent products should be resolved properly by conducting adequate research on this hopeful medicinal plant. If these issues are properly resolved, this medicinal plant can be used as a safe, effective, and affordable form of health care.

## Figures and Tables

**Table 1:** Botanical morphology of *Heliotropium indicum* L.

Habitat	The disturbed areas are garden or lawns, roadsides, anthropogenic habitats, and waste places. It is mostly found at a 1,000 m altitude.	

Foliage	Leaves	4–10 cm long and 2–5 cm wide, opposite, or sub-opposite, alternate or sub-alternate, ovate to obovate, and acute, with a wavy or undulate, serrulate, or cordate leaf margin, nerves on either side or veins. The leaf surface is covered with short hairs, which may be quite stiff.
	Petiole	1–7 cm long with a sub-truncate base or ovate

Flowers	4–5 mm wide, regular, sessile, axillary, and slightly purple or white or whitish violet with a small yellow center and having a narrow tube with lobes formed a plate shape	
	Inflorescence	String or twisted of beads with a prominent curl at the apex. Flowers develop apically within the cymose inflorescence.
	Sepals	5 in number, 3 mm long, diffused with hairs outside, deep green in color, linear to lanceolate, and uneven or unequal
	Calyx lobes ciliate	3 mm long
	Stamens	5 in number and borne in a corolla tube, terminal, and corolla tube 4–6 mm long
	Petals	Rounded
	Ovary	4 lobed

Fruits	Fruits, also known as nutlets, are dry, indehiscent 2–4 lobed, 3–6 mm long, with or without united nutlets, ovate, and ribbed separated into two nutlets. Each nutlet is two-celled and beaked.	

Stem and roots	Wide distributed, branched or unbranched, and hirsute with hairs in the stem. The root system is a long taproot and highly branched.	

Genetics	2*n* = 22, 24	

**Table 2:** Traditional uses of *H. indicum* L.

Country	Local names	Traditional use as or to treat	Part(s) used	Mode of administration	Reference(s)
Bangladesh	Hatisur	Antidote to poisoning	Leaves and stem	Decoction of leaves and stems is administered orally.	[12]
		Swelling of knees, joint pain, and severe itching in leg	Root	Decoction or maceration of the root is used through vocal order (VO).	[25]
		Chicken pox	Leaves	Juice of roots is taken orally.	[26]
		Allergy	Leaves	Juice of the leaf is taken orally.	
		Blood purification and infections	Root	Juice of roots is used both orally and topically.	[27]
Brazil	Aguará-ciunhá-ac¸ú and jacuá-acanga	Skin ulcers and burns	Leaves	Unknown	[19,37]
Benin	Koklosoudèn	Dystocia	Leaves	Trituration with water and drops in eyes	[38]
		Femal	Leaves	Leaf extract is filtered then applied through VO.	
		Leucorrhoea	Whole plant	The diluted juice is administered through VO.	
		Splenomegalia	Leaves	Unknown	
		Psychosis	Leaves and root	Unknown	
	Koclossoudinkpatcha (Fon)	Internal infection and hypertension	Stem and leaves	Decoction of stems with leaves is applied through VO.	[39]
Congo	Not registered	Stomach, fever, and eye lotion	Leaves	Decoction of fresh leaves with water that is taken 1 glass/day for 1 week.	[40]
Colombia	Rabo de alacrán and verbena	Internal parasites	Leaves	Decoction of fresh leaves	[41]
Guinea	Nasinko and hogghonhwan	Diarrhea and febrifuge	Whole plant	Decoction of the whole plant	[42]
		Antiseptic	Leaves	The decoction of leaves is allowed to administer through vocal order.	[43]
Ghana	Kɔmfemtikorɔ	Paludism and eye infections	Leaves	Decoction of leaves is used for 7 days.	[44]
Conakry	Not registered	Fever	Whole plant	Decoction of the whole plant	[9]
Gabon	(mo-)nyaka (w-)a mbumba (Eviya language)	Gingivitis	Leaves	Ground leaves of *H. indicum* for local application	[45]
India	Nakkipoo	Snakebite and scorpion sting	Leaves	The leaf juice is used by mixing with hot water.	[29]
	Indian heliotrope and hatisundha	Wounds and skin infections	Whole plant	Paste of the whole plant is applied topically.	[28]
		Ophthalmia	Root	Juice of the root is taken orally.	[46]
Ivory Coast	Klaouri (Gouro), kotokorokombo (Baoule), nansifo, nosiko (Malinke), tapentiti, and taperodia (Shien)	Colds and sinusitis	Leaves	Powder of dry leaves	[47]
Indonesia	Bandotanlombok, djingirajam, gadjahan, tlale, and tusokkonde	Herpes and rheumatism	Leaves	Decoction of leaf is used in thrush and poultices.	[9]
Jamaica	Turnsoles	Menorrhagia	Flower	Infusion of the flower is taken orally.	[48]
		Fever, ulcers, venereal diseases, and sore throat	Whole plant	Decoction of the whole plant is taken orally.	
		Induced abortion	Whole plant	Decoction of the whole plant is applied to the vaginal cavity.	
		Rectal sores	Whole plant	Decoction of the whole plant is administered rectally.	
		Cleansing and dressing of wounds and ulcers	Whole plant	Paste of fresh plant	
Mauritius	Herbepapillon (Creole) and taylkoudougou (Tamoul)	Renal colic	Leaves	Infusion of 4 or 5 green leaves	[32,49]
		Ophthalmia, diuretic, anthrax (poultice), and ulcers	Leaves	Diluted leaf of 1 or 2 cups	
Mali	Nonsikou (Bambara)	Nausea and vomiting	Whole plant	Boiled decoction of plant bundle is taken orally.	[50]
		Baby thinness	Leaves	Leaves decoction through VO and bath 4x/day for 10 days	[9]
		Ocular infection	Leaves	Leaves decoction is used to wash eyes.	
		Amenorrhea	Root	Decoction of roots is applied through VO and bath for 3 days.	
		High blood pressure	Leaves	Leaves decoction (VO)	
Mexico	Not registered	Asthma	Root	Decoction of roots or any plant part	[9]
Nigeria	Agogo-igun, ogbe, and akuko	Paludism, and sap is applied to gumboils.	Leaves	The decoction with water and allowed to administer through vocal order	[38]
		Hepatitis and fever	Leaves	The decoction with water and allowed to administer through vocal order	
		Gonorrhea	Leaves	The leaf juice mixed with castor oil is locally applied.	[51]
	Otukeyin, ekaesinono, and edisimon (Ibibio)	Boils and sore throat	Leaves	Decoction of crushed leaves is applied through VO.	[52]
Nicaragua	Not registered	Skin infections	Leaves	Leaf paste is applied topically for skin infections.	[53,54]
		Whooping cough	Leaves and root	Decoction of a combination of leaf and root is taken orally.	
Philippines	Buntot-leon, pengnga-pengnga, and puntaelepante	Diuretic and kidney stone	Whole plant	Decoction of the whole plant is taken orally.	[55]
Rodrigues Island	Herbepapillon	Calculus	Whole plant	Decoction of the plant is applied through VO.	[56]
	Herbepapillon (Rodrigues Creole) and Indian heliotrope (English)	Bloating and loss of appetite	Leaves	Decoction of the leaves (VO). 1 cup when needed.	[57]
Siby	Nonsikou	Vomiting	Leaves	Unknown	[9]
Seychelles	Not registered	Chirurgical pain	Leaves	The decoction with water and allowed to administer through vocal order	[58]
Senegal	Manding-bambarańâgiku	Child, eczema, impetigo, and dermatitis	Leaves	The leaf powder is prepared by drying in the shadow and in the open-air that is applied in local.	[59]
		Diuretic and kidney stone	Whole plant	Decoction of the whole plant is taken orally.	[55,60]
Sao Tome	Folhagalo	Ulcers	Leaves	The crushed leaves with palm oil are applied on the affected area.	[61]
Sierra Leone	Not registered	Washing the newborn babies	Leaves	Decoction of leaves	[9]
South America	Not registered	Insect bites and scorpion stings	Leaves and root	Paste of leaf and root together is applied externally.	[62]
Togo	Koklotadoe and agamassiké (Ewé)	Dermatosis	Leaves	Local application of leaves juice	[38]
		Liver diseases	Whole plant	Decoction of the whole plant	[63]
Tanzania	Humbangara (Ngoni)	Yaws	Root	Decoction or maceration of the root through VO	[64]
Taiwan	Gou-wei-chung-tsan	Hepatitis	Leaves and root	Paste of leaf and root together is applied externally.	[65]
Thailand	Yah nguang-chang	Produce permanent sterilization in females	Inflorescence	One gram of the dried and powdered inflorescence mixed with milk or water is used for 3 days beginning with the fourth day of menses to achieve the desired result.	[33]
West Indies		Head lice	Whole plant	Paste of fresh whole plant	[66]

**Table 3:** Chemical compounds isolated from *H. indicum* L.

Phytochemicals	Part(s)	Reference(s)
*Alkaloids*		
Cynoglossine	Seed	[67]
Echinatine	Aerial	[53]
Heleurine	Aerial	[62]
Heliotrine	Aerial	[62]
Heliotridine	Aerial	[62]
Helindicine	Root	[75]
Europine *N*-oxide	Seed	[67]
Heleurine *N*-oxide	Seed	[67]
Heliotridine *N*-oxide	Seed	[67]
Heliotrine *N*-oxide	Seed	[67]
Indicine	Aerial	[53]
Indicine *N*-oxide	Aerial	[71]
Lasiocarpine	Aerial	[65]
Lycopsamine	Root	[75]
Trachelanthamidine	Leaves	[76]
Retronecine	Leaves and aerial	[73,76]
Supinine	Aerial	[53]
Triterpenes		
*β*-Amyrin	Whole plant	[77]
Lupeol	Aerial and whole plant	[71,77]
Rapanone	Whole plant	[77]

*Sterols*		
*β*-Sitosterol	Whole plant	[77]
Estradiol	Root	[78]
Chalinasterol	Whole plant	[77]
Campesterol	Whole plant	[77]
Hexacosane-1-ol	Whole plant	[77]
Stigmasterol	Whole plant	[77]

*Amines*		
Putrescine	Leaves	[76]
Spermidine	Leaves	[76]
Spermine	Leaves	[76]

*Volatile oils*		
1-Dodecanol	Whole plant	[79]
*β*-Linalool	Whole plant	[79]
Phytol	Whole plant	[79]

**Table 4:** Pharmacological activities of different parts of *H. indicum* L.

Activity	Extract	Method	Results	References
Antioxidant activity	Methanolic extract of leaf, stem, and root	DPPH free radical scavenging assay	Leaf extract yields greater free radical scavenging activity than the stem and roots.	[80]
	Aqueous leaf extract		Show high free radical scavenging activity compared with *Centella asiatica, Coccinia grandis,* and *Euphorbia hirta.*	[81]
	Ethanol and water extracts of the whole plant		Ethanolic extract showed high antioxidant activity.	[14]
Analgesic activity	Aqueous and ethanol extracts of the whole plant	*In vivo*: Formalin-induced nociception in mice	Both extracts have analgesic activity.	[82]
Antinociceptive activity	Methanolic extract of the roots	Acetic-acid-induced writhing in mice	Extract produced writhing inhibition in the test animals.	[7]
	Chloroform extract of leaves	Hot-plate model in male Swiss albino mice	Extract showed writhing inhibition in mice.	[83]
Antiinflammatory activity	Methanol extracts of leaf, stem, and root	Egg-albumin- and carrageenin-induced acute paw edema models and cotton pellet granuloma sub-acute inflammation model	Extract of roots produced a significant antiinflammatory effect in acetic-acid-induced writhing in mice.	[18,84]
	Chloroform extract of leaves	Carrageenan-induced raw paw edema	The extract showed maximum inhibition on carrageenan-induced rat paw edema.	[83]
	Aqueous whole plant extract	Lipopolysaccharide (LPS) induced uveitis rabbits	The extract reduced both the clinical scores of inflammation and inflammatory cells infiltration.	[85]
Antimicrobial activity	Alcoholic extract of the whole plant	Agar cup plate diffusion method	The alcoholic extract was found to possess dose-dependent antimicrobial activity against bacteria, fungi, and yeasts,	[8,86]
	Petroleum ether, chloroform, aqueous, and methanolic extracts of leaves		All extracts show effective antimicrobial activity against both Gram-positive and Gram-negative bacteria	[6,37]
	Aqueous, ethanol, and chloroform extracts of the whole plant		Had significant zones of inhibition against bacteria and fungi.	[87]
	Methanol whole plant extracts		Exhibited both antibacterial and antifungal activity.	[88]
	Methanol leaves extract		Had an antibacterial activity.	[8]
Antituberculosis activity	Volatile oil of *H. indicum* from aerial parts	Alamar blue assay system with an MIC	Had profound antituberculosis activity against *Mycobacterium tuberculosis* H37Ra.	[79]
Antihyperglycemic activity	Whole plant methanol extracts	Tested on the fasting blood glucose levels of streptozotocin-induced (STZ-induced) diabetic rats	Showed a conspicuous reduction in blood glucose levels and normalization of blood glucose levels.	[89]
Anticataract activity	Ethanolic leaf extract	Galactose-induced cataract in rats	Significantly increased the lens glutathione.	[20]
	Aqueous extract of the whole plant	Selenite-induced cataracts in Sprague–Dawley rats	Expressively inhibited the development of selenite-induced cataracts.	[90]
Antiplasmodial properties	Dichloromethane, methanol, and total aqueous extracts of the whole plant	Tested on chloroquine-sensitive (3D7) and resistant (W2) strains of *Plasmodium falciparum*	Revealed no direct antiplasmodial activity.	[91]
Antifertility activity	Petroleum ether extract of the whole plant	*In vivo* test on rats	Exhibited profound activity.	[77]
	Extract of the n-hexane and benzene fractions of whole plant	Antiimplantation and abortifacient models in rats	Had substantial antifertility activity.	[92]
Anthelmintic activity	Methanolic extract of leaves	*In vitro* anthelmintic bioassay	The extract showed significant anthelmintic efficacy.	[93]
Antitumor activity	Methanolic extract of both stem and leaf	MTT assay on HeLa cell lines	Both extracts exhibited antiproliferative activity where the stem extract showed interesting results.	[94]
	Ethanolic extract of the whole plant	MTT assay on SKBR3 human breast adenocarcinoma cell line	Showed momentous antiproliferative activity.	[91]
Antitussive property	Ethanolic extract of leaves	The citric acid saturated chamber in animals	Extract syrup recorded the lowest number of coughs.	[95]
Antiglaucoma activity	Aqueous whole plant extract	Glaucoma of rabbits *in vivo*	Significantly reduced intraocular pressure in acute and chronic glaucoma.	[90]
Wound-healing activity	Dried parts of ethanolic extracts	Excision and restored incision wound model	Showed wound-healing capacity.	[19]
	*n*-Butanol fractions aerial part (stem and leaves)	The scratch assay	The isolated compound contains profound wound-healing activity.	[96]
	The petroleum ether, chloroform, methanol, and aqueous extracts of leaves.	Excision (normal and infected), incision, and dead space wound models in rats	Methanol and aqueous extracts attributed intense wound-healing activity.	[37]
Histo-gastroprotective activity	Aqueous extract of the dried leaves	Indomethacin-induced gastric ulcerated mucosa in rats	Had effective histo-gastroprotective activity.	[10]
Diuretic activity	Methanolic extract of the dried roots	Biuret, a urea derivative assayed by the electrolyte loss ratio (Na^+^/K^+^ excretion ratio) in mice	The extract revealed a marked diuretic effect.	[7]
Relaxant/receptor property	Ethanol extract of the roots	Guinea pig ileum and rabbit duodenum *in vitro*	Possess weak smooth muscle relaxant activity.	[97]
	Dark-brown solid extract of aerial parts	Guinea pig ileum, rabbit jejunum, rat uterus, and rat anococcygeus preparations *in vivo*	Showed profound receptor property	[98]
Clot lysis and membrane-stabilizing activities	Ethanolic, petroleum ether, carbon tetrachloride, and chloroform extracts of leaves	Membrane-stabilizing and thrombolytic activities *in vitro*	Had potential clot lysis and membrane-stabilizing activities.	[99]
	Methanol extract of the whole plant	*In vitro* thrombolytic model and membrane-stabilizing activity assay on human RBC subjected to heat and hypotonic stress	Protected the hemolysis of RBCs induced by hypotonic solution and heat stress.	[88]
Antiallergic activity	Aqueous whole plant extract	Ovalbumin-induced allergic conjunctivitis on Dunkin–Hartley guinea pigs	Exhibited antiallergic effect possibly by immunomodulation or immunosuppression.	[90]
Larvicidal activity	Ethanolic leaf extract	Larvicidal bioassay on mosquito larvae of *Aedes aegypti*	The extract showed effective mosquito larvicidal activity.	[100]
Pesticidal activity	Ethanol extract of aerial parts	Brine shrimp lethality bioassay	Possess potent activity against the brine shrimp nauplii.	[7]

## Abbreviations

AA: Ascorbic acid
BHT: Butylated hydroxytoluene
DPPH: 1-Diphenyl-2 picrylhydrazyl
EC_50_: Half-maximal effective concentration
GC-FID: Gas chromatography–flame ionization detector
GC-MS: Gas chromatography–mass spectrometry
IC_50_: Half-maximal inhibitory concentration
LPS: Lipopolysaccharide
MIC: Minimum inhibitory concentration
mTOR: Mammalian target of rapamycin
NMR: Nuclear magnetic resonance
RBC: Red blood cell
STZ: Streptozotocin
VO: Vocal order
WHO: World Health Organization.

## References

[1] Mukherjee P. K., Venkatesh P., Ponnusankar S. (2010). Ethnopharmacology and integrative medicine-let the history tell the future. *Journal of Ayurveda and Integrative Medicine*.

[2] Calixto J. B., Santos A. R., Cechinel Filho V., Yunes R. A. (1998). A review of the plants of the genus *Phyllanthus*: their chemistry, pharmacology, and therapeutic potential. *Medicinal Research Reviews*.

[3] Sharifi-Rad M., Lankatillake C., Dias D. A. (2020). Impact of natural compounds on neurodegenerative disorders: from preclinical to pharmacotherapeutics. *Journal of Clinical Medicine*.

[4] Fabricant D. S., Farnsworth N. R. (2001). The value of plants used in traditional medicine for drug discovery. *Environmental Health Perspectives*.

[5] Salehi B., Calina D., Docea A. O. (2020). Curcumin’s nanomedicine formulations for therapeutic application in neurological diseases. *Journal of Clinical Medicine*.

[6] Oluwatoyin S., Ndukwe G. I., Joseph A. (2011). Phytochemical and antimicrobial studies on the aerial parts of *Heliotropium indicum* Linn. *Annals of Biological Research*.

[7] Rahman M. A., Mia M., Shahid I. (2011). Pharmacological and phytochemical screen activities of roots of *Heliotropium indicum* Linn. *PharmacologyOnLine*.

[8] Osungunna M. O., Adedeji K. A. (2016). Phytochemical and antimicrobial screening of methanol extract of *Heliotropium indicum* leaf. *Journal of Microbiology and Antimicrobials*.

[9] Togola A., Diallo D., Dembélé S., Barsett H., Paulsen B. S. (2005). Ethnopharmacological survey of different uses of seven medicinal plants from Mali, (West Africa) in the regions Doila, Kolokani and Siby. *Journal of Ethnobiology and Ethnomedicine*.

[10] Adelaja A. A., Ayoola M. D., Otulana J. O., Akinola O. B., Olayiwola A., Ejiwunmi A. B. (2008). Evaluation of the histo - gastroprotective and antimicrobial activities of *Heliotropium indicum* Linn (boraginaceae). *Malaysian Journal of Medical Sciences*.

[11] Ayyanar M., Ignacimuthu S. (2009). Herbal medicines for wound healing among tribal people in Southern India: ethnobotanical and scientific evidences. *International Journal of Applied Research in Natural Products*.

[12] Nawaz A. H., Hossain M., Karim M., Khan M., Jahan R., Rahmatullah M. (2009). An ethnobotanical survey of Rajshahi district in Rajshahi division, Bangladesh. *American-Eurasian Journal of Sustainable Agriculture*.

[13] Dash G. K., Abdullah M. S. (2012). A review on *Heliotropium indicum* L. (Boraginaceae). *International Journal of Pharmaceutical Sciences and Research*.

[14] Chunthorng-Orn J., Dechayont B., Phuaklee P., Prajuabjinda O., Juckmeta T., Itharat A. (2016). Cytotoxic, anti-inflammatory and antioxidant activities of *Heliotropium indicum* extracts. *Journal of the Medical Association of Thailand*.

[15] Kugelman M., Liu W. C., Axelrod M., McBride T. J., Rao K. V. (2015). Indicine-*N*-oxide: the antitumor principle of *Heliotropium indicum*. *Lloydia*.

[16] Schoental R. (1968). Toxicology and carcinogenic action of pyrrolizidine alkaloids. *Cancer Research*.

[17] Hartmann T., Ober D. (2000). *Biosynthesis and Metabolism of Pyrrolizidine Alkaloids in Plants and Specialized Insect Herbivores*.

[18] Srinivas K., Rao M. E. B., Rao S. (2000). Anti-inflammatory activity of *Heliotropium indicum* Linn and *Leucas aspera* Spreng. in albino rats. *Indian Journal of Pharmacology*.

[19] Reddy J. S., Rao P. R., Reddy M. S. (2002). Wound healing effects of *Heliotropium indicum, Plumbago zeylanicum* and *Acalypha indica* in rats. *Journal of Ethnopharmacology*.

[20] Veda V. T., Sasi K. S., Asokan B. R., Sengottuvelu S., Jaikumar S. (2016). Anticataract activity of ethanolic extract of *Heliotropium indicum* leaves on galactose induced cataract in rats. *International Journal of Pharmacology & Toxicology*.

[21] Kandemir N., Çelik A., Shah S. N., Razzaq A. (2020). Comparative micro-anatomical investigation of genus *Heliotropium* (Boraginaceae) found in Turkey. *Flora*.

[22] Ghosh P., Das P., Das C., Mahapatra S., Chatterjee S. (2018). Morphological characteristics and phytopharmacological detailing of hatishur (*Heliotropium indicum* Linn.): a concise review. *Journal of Pharmacognosy and Phytochemistry*.

[23] Reyes-García V. (2010). The relevance of traditional knowledge systems for ethnopharmacological research: theoretical and methodological contributions. *Journal of Ethnobiology and Ethnomedicine*.

[24] Nisar M. F., Jaleel F., Waseem M., Ismail S., Toor Y., Mujtaba Haider S. (2014). Ethno-medicinal uses of plants from district Bahawalpur, Pakistan. *Current Research Journal of Biological Sciences*.

[25] Kamal Z., Bairage J., Moniruzzaman (2014). Ethnomedicinal practices of a folk medicinal practitioner in Pabna district, Bangladesh. *World Journal of Pharmacy and Pharmaceutical Sciences*.

[26] Shahnaj S., Asha U., Mim T. (2015). A survey on the ethnomedicinal practices of a folk medicinal practitioner in Manikganj district, Bangladesh. *Journal of Chemical and Pharmaceutical Research*.

[27] Akhter J., Khatun R., Akter S. (2021). Ethnomedicinal practices in Natore district, Bangladesh. *World Journal of Pharmacy and Pharmaceutical Sciences*.

[28] Muthu C., Ayyanar M., Raja N., Ignacimuthu S. (2006). Medicinal plants used by traditional healers in Kancheepuram district of Tamil Nadu, India. *Journal of Ethnobiology and Ethnomedicine*.

[29] Alagesaboopathi C. (2009). Ethnomedicinal plants and their utilization by villagers in Kumaragiri hills of Salem district of Tamilnadu, India. *African Journal of Traditional, Complementary and Alternative Medicines*.

[30] Wiart C. (2006). *Medicinal Plants of the Asia-Pacific*.

[31] Odugbemi T. O., Akinsulire O. R., Aibinu I. E., Fabeku P. O. (2007). Medicinal plants useful for malaria therapy in Okeigbo, Ondo state, Southwest Nigeria. *African Journal of Traditional, Complementary and Alternative Medicines*.

[32] Suroowan S., Pynee K. B., Mahomoodally M. F. (2019). A comprehensive review of ethnopharmacologically important medicinal plant species from Mauritius. *South African Journal of Botany*.

[33] Berhaut J. Flore illustrée du Sénégal. *Gouvernement du Sénégal, Ministère du développement rural, Direction des eaux et forêts*.

[34] Dey K. L. (1896). *The Indigenous Drugs of India*.

[35] Chopra R. N., Nayar S. L., Chopra I. C. (1956). *Glossary of Indian Medicinal Plants*.

[36] Dahanukar S. A., Kulkarni R. A., Rege N. N. (2000). Pharmacology of medicinal plants and natural products. *Indian Journal of Pharmacology*.

[37] Dash G. K., Murthy P. N. (2011). Studies on wound healing activity of *Heliotropium indicum* Linn. leaves on rats. *ISRN Pharmacology*.

[38] Adjanohoun E. (2014). Le processus de rénovation de la pharmacopée africaine. *Bulletin de la Société Botanique de France Actualités Botaniques*.

[39] Apema R., Mozouloua D., Kosh-Komba E., Ngoule Y. Les plantes médicinales utilisées dans le traitement de l’hypertension artérielle par les tradipraticiens à Bangui.

[40] Kalanda K., Omasombo W. D. (2019). Contribution à la connaissance des plantes médicinales du Haut Zaïre: plantes utilisées dans le traitement des maux d’estomac dans la ville de Kisangani. *Revue de Médecine et de Pharmacie*.

[41] Agudelo-Lopez S., Gomez-Rodriguez L., Coronado X. (2008). [Prevalence of intestinal parasitism and associated factors in a village on the Colombian Atlantic Coast]. *Revista de Salud Pública*.

[42] Carrière M. Plantes de Guinée à l’usage des éleveurs et des vétérinaires. Annexes..

[43] Magassouba F. B., Diallo A., Kouyate M. (2007). Ethnobotanical survey and antibacterial activity of some plants used in Guinean traditional medicine. *Journal of Ethnopharmacology*.

[44] Komlaga G., Agyare C., Dickson R. A. (2015). Medicinal plants and finished marketed herbal products used in the treatment of malaria in the Ashanti region, Ghana. *Journal of Ethnopharmacology*.

[45] Walker R. (1986). Usages pharmaceutiques des plantes spontanées du Gabon. *Institut d’Études Centrafricaines*.

[46] Das A. K., Dutta B. K., Sharma G. D. (2008). Medicinal plants used by different tribes of Cachar district, Assam. *Indian Journal of Traditional Knowledge*.

[47] Bouquet A. (1974). Plantes médicinales de la Côte d’Ivoire. *ORSTOM*.

[48] Asprey G. F., Thornton P. (1955). Medicinal plants of Jamaica. Parts III. *West Indian Medical Journal*.

[49] Daruty C. (2018). *Plantes médicinales de I’lle Maurice et des pays intertropicaux*.

[50] Nordeng H., Al-Zayadi W., Diallo D., Ballo N., Paulsen B. S. Traditional medicine practitioners’ knowledge and views on treatment of pregnant women in three regions of Mali. *Journal of Ethnobiology and Ethnomedicine*.

[51] Ainslie J. R. *The List of Plants Used in Native Medicine in Nigeria, Imp*.

[52] Ajibesin K. K., Ekpo B. A., Bala D. N., Essien E. E., Adesanya S. A. (2008). Ethnobotanical survey of Akwa Ibom state of Nigeria. *Journal of Ethnopharmacology*.

[53] Coe F. G., Anderson G. J. (1996). Ethnobotany of the garífuna of Eastern Nicaragua. *Economic Botany*.

[54] Barrett B. (1994). Medicinal plants of Nicaragua’s atlantic coast. *Economic Botany*.

[55] Quisumbing E. Medicinal plants of the Philippines. *Tech Bull*.

[56] Gurib-Fakim A., Gueho J., Sewraj-Bissoondoyal M. (2008). The medicinal plants of Mauritius–part 1. *International Journal of Pharmacognosy*.

[57] Samoisy A. K., Mahomoodally M. F. (2015). Ethnopharmacological analysis of medicinal plants used against non-communicable diseases in Rodrigues Island, Indian Ocean. *Journal of Ethnopharmacology*.

[58] Adjanohoun E. J., Abel A., Aké Assi L. Médecine traditionelle et pharmacopée-contribution aux études ethnobotaniques et floristiques aux Seychelles.

[59] Kerharo J., Adam J. G. (1974). La pharmacopée sénégalaise traditionnelle: plantes médicinales et toxiques. *Editions Vigot Frères*.

[60] Berhault J. Floore Illustree du Senegal. *Govt Senegal, Min Rural Development, Water and Forest Division, Dakar*.

[61] Sequeira V. Medicinal plants and conservation in São Tomé. *Biodiversity & Conservation*.

[62] Duke J. A. (1994). *Amazonia Ethnobotanical Dictionary*.

[63] Kpodar M. S., Karou S. D., Katawa G. (2015). An ethnobotanical study of plants used to treat liver diseases in the maritime region of Togo. *Journal of Ethnopharmacology*.

[64] Kokwaro J. O. (1976). Medicinal plants of east Africa. *East African Literature Bureau*.

[65] Lin C. C., Kan W. S. (1990). Medicinal plants used for the treatment of hepatitis in Taiwan. *The American Journal of Chinese Medicine*.

[66] Ayensu E. S. (1981). *Medicinal Plants of the West Indies*.

[67] Williaman J. J., Schubert B. G. (1962). Alkaloid-bearing plants and their contained alkaloids. Technical Bulletin No. 1234. *Journal of Pharmaceutical Sciences*.

[68] Mattocks A. R., Schoental R., Crowley H. C., Culvenor C. C. J. (1961). Indicine: the major alkaloid of *Heliotropium indicum* L. *Journal of the Chemical Society*.

[69] Hoque M. S., Ghani A., Rashid H. (1976). Alkaloids of *Heliotropium indicum* L. grown in Bangladesh. *Bangladesh Pharmaceutical Journal*.

[70] Pandey V. B., Singh J. P., Rao Y. V., Acharya S. B. (1982). Isolation and pharmacological action of heliotrine, the major alkaloid of *Heliotropium indicum* seeds. *Planta Medica*.

[71] Pandey D. P., Singh J. P., Roy R., Singh V. P., Pandey V. B. (1996). Constituents of *Heliotropium indicum*. *Oriental Journal of Chemistry*.

[72] Sivagnanam S., Singh M. K., Satish M. K., Rao M. R. K. (2014). Preliminary phytochemical analysis of *Amaranthus* polygonoides. *Research Journal of Pharmaceutical, Biological, and Chemical Sciences*.

[73] Mattocks A. R. (1967). Minor alkaloids of *Heliotropium indicum* L. *Journal of the Chemical Society C: Organic*.

[74] Birecka H., Frohlich M. W., Glickman L. M. (1983). Free and esterified necines in *Heliotropium* species from Mexico and Texas. *Phytochemistry*.

[75] Souza J. S. N., Machado L. L., Pessoa O. D. L. (2005). Pyrrolizidine alkaloids from *Heliotropium indicum*. *Journal of the Brazilian Chemical Society*.

[76] Birecka H., DiNolfo T. E., Martin W. B., Frohlich M. W. (1984). Polyamines and leaf senescence in pyrrolizidine alkaloid-bearing *Heliotropium* plants. *Phytochemistry*.

[77] Andhiwal C. K., Has C., Varshney R. P. (2013). Chemical and pharmacological studies of *Heliotropium indicum*. *Indian Drugs*.

[78] Mannan A., Ahmad K. (1978). Preliminary study of sex hormones of medical importance in Bangladeshi plants. *Bangladesh Medical Research Council Bulletin*.

[79] Machan T., Korth J., Liawruangrath B., Liaewruangrath S., Pyne S. (2005). Composition and antituberculosis activity of the volatile oil of *Heliotropium indicum* Linn. growing in Phitsanulok, Thailand. *Flavour and Fragrance Journal*.

[80] Kumar M. S., Chaudhury S., Balachandran S. (2014). In vitro callus culture of *Heliotropium indicum* Linn. for assessment of total phenolic and flavonoid content and antioxidant activity. *Applied Biochemistry and Biotechnology*.

[81] Kumar M., Kumar S., Balachandran S., Chaudhury S. (2012). Influence of incubation temperatures on total phenolic, flavonoids content and free radical scavenging activity of callus from *Heliotropium indicum* L. *Asian Journal of Pharmaceutical Research*.

[82] Boye A., Koffuor G. A., Amoateng P., Ameyaw E. O., Abaitey A. K. (2012). Analgesic activity and safety assessment of *Heliotropium indicum* Linn. (Boraginaceae) in rodents. *International Journal of Pharmacology*.

[83] Betanabhatla K. S., Jasmin S. R., Raamamurthy J., Christina A. J., Sasikumar S. (2007). Anti-inflammatory and anyinociceptive activities of *Heliotropium indicum* Linn. experimental animal models. *PharmacologyOnLine*.

[84] Shalini S., Kaza R., Shaik F. (2010). Study on the anti-inflammatory activity of *Heliotropium indicum*. *Journal of Innovative Trends in Pharmaceutical Sciences*.

[85] Kyei S., Koffuor G. A., Ramkissoon P., Ameyaw E. O., Asiamah E. A. (2016). Anti-inflammatory effect of *Heliotropium indicum* Linn on lipopolysaccharide-induced uveitis in New Zealand white rabbits. *International Journal of Ophthalmology*.

[86] Rao P. R., Nammi S., Raju A. D. V. (2002). Studies on the antimicrobial activity of *Heliotropium indicum* Linn. *Journal of Natural Remedies*.

[87] Premnath D., Gomez P. (2012). Antifungal and anti bacterial activities of chemical constituents from *Heliotropium indicum* Linn. Plant. *Drug Invention Today*.

[88] Mourin N. A., Sharmin T., Chowdhury S. R., Islam F., Rahman M. S., Rashid M. A. (2013). Evaluation of bioactivities of *Heliotropium indicum*, a medicinal plant of Bangladesh. *Pharma Innovation*.

[89] Mohammad S. A., Abdul Nabi S., Marella S. (2015). Phytochemical screening and antihyperglycemic activity of *Heliotropium indicum* whole plant in streptozotocin induced diabetic rats. *Journal of Applied Pharmaceutical Science*.

[90] Kyei S., Koffuor G. A., Ramkissoon P., Afari C., Asiamah E. A. (2015). The claim of anti-cataract potential of *Heliotropium indicum*: a myth or reality?. *Ophthalmology and Therapy*.

[91] Goyal N., Sharma S. (2014). Bioactive phytoconstituents and plant extracts from genus *Heliotropium*. *International Journal of Green Pharmacy*.

[92] Savadi R. V., Alagawadi K. R., Darade S. S. (2009). Antifertility activity of ethanolic extract and its n-hexane and benzene fractions of *Heliotropium indicum* leaves on albino rats. *Journal of Pharmacy Research*.

[93] Mahato K., Kakoti B. B., Borah S., Kumar M. (2014). Evaluation of *in-vitro* anthelmintic activity of *Heliotropium indicum* Linn. leaves in Indian adult earthworm. *Asian Pacific Journal of Tropical Disease*.

[94] Sivajothi V., Shruthi D. S., Sajini J. R. (2015). Cytotoxic effect of *Heliotropium indicum* extracts on Hela cell line. *International Journal of Pharmacy and Pharmaceutical Sciences*.

[95] Villa M. H., Peria J. N. T., Mangansat N. J. M., Dulay R. M. R. (2016). Antitussive and antibacterial activity of *Trompang elepante* (*Heliotropium indicum* Linn.). *Asian Journal of Plant Science and Research*.

[96] Dodehe Y., Barthelemy A., Calixte B., Jean D. N., Allico J. D., Nelly F. (2013). *In vitro* wound healing effect of n-butanol fractions from *Heliotropium indicum*. *Just-in-Time Production Systems*.

[97] Vieira J. E. V., Barros G. S. G., Medeiros M. C., Matos F. J. A., Souza M. P., Medeiros M. J. (1972). Pharmacologic screening of plants from Northeast Brazil. II. *Revista brasileira de farmácia*.

[98] Koffuor G. A., Boye A., Amoateng P., Ameyaw E. O., Abaitey A. K. (2012). Investigating the site of action of an aqueous extract of *Heliotropium indicum* Linn (Boraginaceae) on smooth muscles. *Research Journal of Pharmacology*.

[99] Samira K., Laboni F. R., Julie A. S., Jalal U., Labu Z. K. (2016). Biological investigations of medicinal plants of *Heliotropium indicum* indigenous to Bangladesh. *Journal of Coastal Life Medicine*.

[100] Ramamurthy V., Krishnaveni S. (2014). Larvicidal efficacy of leaf extracts of *Heliotropium Indicum* and *Mukia maderaspatana* against the dengue fever mosquito vector *Aedes aegypti*. *Journal of Entomology and Zoology Studies*.

[101] Roy A. (2015). Pharmacological activities of Indian Heliotrope (*Heliotropium indicum* L.): a review. *Journal of Pharmacognosy and Phytochemistry*.

[102] Pianowski L. F., Calixto J. B., Chaves C. P. (2011). *Pharmaceutical Oral Product Obtained from Parts of Heliotropium Plants*.

[103] Bero J., Ganfon H., Jonville M. C. (2009). *In vitro* antiplasmodial activity of plants used in Benin in traditional medicine to treat malaria. *Journal of Ethnopharmacology*.

[104] Ohnuma T., Sridhar K. S., Ratner L. H., Holland J. F. (1982). Phase I study of indicine *N*-oxide in patients with advanced cancer. *Cancer Treatment Reviews*.

[105] Bose A., Mondal S., Gupta J., Dash G., Ghosh T., Si S. (2006). Studies on diuretic and laxative activity of ethanolic extract and its fractions of *Cleome rutidosperma* aerial parts. *Pharmacognosy Magazine*.

[106] Veerakumar K., Govindarajan M., Rajeswary M., Muthukumaran U. (2014). Mosquito larvicidal properties of silver nanoparticles synthesized using *Heliotropium indicum* (Boraginaceae) against *Aedes aegypti, Anopheles stephensi*, and *Culex quinquefasciatus* (Diptera: Culicidae). *Parasitology Research*.

[107] Barros G. S., Matos F. J., Vieira J. E., Sousa M. P., Medeiros M. C. (1970). Pharmacological screening of some Brazilian plants. *Journal of Pharmacy and Pharmacology*.

[108] Kumarasamyraja D., Jeganathan N. S., Manavalan R. (2012). A review on medicinal plants with potential wound healing activity. *International Journal of Pharmacy and Pharmaceutical Sciences*.

[109] Christie G. S., Le Page R. N. (1962). Liver damage in acute heliotrine poisoning. 1. The intracellular distribution of pyridine nucleotides. *Biochemical Journal*.

[110] Rao M. S., Reddy J. K. (1978). Malignant neoplasms in rats fed lasiocarpine. *British Journal of Cancer*.

[111] Armstrong S. J., Zuckerman A. J. (1972). The effects of lasiocarpine, retrorsine and retronecine pyrrole on human embryo lung and liver cells in culture. *British Journal of Experimental Pathology*.

[112] Owolabi M. A., Oribayo O. O., Ukpo G. E., Mbaka G. O., Akindehin O. E. (2015). A 5-month toxicity study of the ethanol extract of the leaves of *Heliotropium indicum* in Sprague Dawley rats after oral administration. *Nigerian Quarterly Journal of Hospital Medicine*.

[113] Mattocks A. R. (1968). Toxicity of pyrrolizidine alkaloids. *Nature*.

[114] Moreira R., Pereira D. M., Valentao P., Andrade P. B. (2018). Pyrrolizidine alkaloids: chemistry, pharmacology, toxicology and food safety. *International Journal of Molecular Sciences*.

[115] Zhao Y., Xia Q., Yin J. J., Lin G., Fu P. P. (2011). Photoirradiation of dehydropyrrolizidine alkaloids--formation of reactive oxygen species and induction of lipid peroxidation. *Toxicology Letters*.

[116] Huxtable R. J. (1989). Human health implications of pyrrolizidine alkaloids and herbs containing them. *Toxicants of Plant Origin*.

[117] Appadurai P., Rathinasamy K. (2013). Indicine *N*-oxide binds to tubulin at a distinct site and inhibits the assembly of microtubules: a mechanism for its cytotoxic activity. *Toxicology Letters*.

[118] Cook B. A., Sinnhuber J. R., Thomas P. J. (1983). Hepatic failure secondary to indicine *N*-oxide toxicity. A pediatric oncology group study. *Cancer*.

[119] Letendre L., Ludwig J., Perrault J., Smithson W. A., Kovach J. S. (1984). Hepatocellular toxicity during the treatment of refractory acute leukemia with indicine *N*-oxide. *Cancer*.

[120] Santos F. A., Frota J. T., Arruda B. R. (2012). Antihyperglycemic and hypolipidemic effects of alpha, beta-amyrin, a triterpenoid mixture from *Protium heptaphyllum* in mice. *Lipids in Health and Disease*.

[121] Saleem M. (2009). Lupeol, a novel anti-inflammatory and anti-cancer dietary triterpene. *Cancer Letters*.

[122] Pardo Andreu G. L., Reis F. Z. D., González-Durruthy M. (2020). Rapanone, a naturally occurring benzoquinone, inhibits mitochondrial respiration and induces HepG2 cell death. *Toxicology in Vitro*.

[123] Thirupathi A., Silveira P. C., Nesi R. T., Pinho R. A. (2016). *β*-Amyrin, a pentacyclic triterpene, exhibits anti-fibrotic, anti-inflammatory, and anti-apoptotic effects on dimethyl nitrosamine-induced hepatic fibrosis in male rats. *Human & Experimental Toxicology*.

[124] Calle J., Olarte J., Pinzon R., Ospina L. F., Mendoza M. C., Orozco M. J. (2000). Alterations in the reproduction of mice induced by rapanone. *Journal of Ethnopharmacology*.

[125] Wróbel-Biedrawa D., Grabowska K., Galanty A., Sobolewska D., Żmudzki P., Podolak I. (2020). Anti-melanoma potential of two benzoquinone homologues embelin and rapanone-a comparative *in vitro* study. *Toxicology in Vitro*.

[126] Hartmann M.-A., Daum G. (2004). 5 Sterol metabolism and functions in higher plants. *Lipid Metabolism and Membrane Biogenesis*.

[127] Ferrer A., Altabella T., Arro M., Boronat A. (2017). Emerging roles for conjugated sterols in plants. *Progress in Lipid Research*.

[128] Stillwell W. (2016). *An Introduction to Biological Membranes*.

[129] Plat J., Baumgartner S., Vanmierlo T. (2019). Plant-based sterols and stanols in health & disease: consequences of human development in a plant-based environment?. *Progress in Lipid Research*.

[130] Wang X., Wu G., Bazer F. W. (2016). mTOR: the master regulator of conceptus development in response to uterine histotroph during pregnancy in ungulates. *Molecules to Medicine with mTOR*.

[131] Minois N., Rockenfeller P., Smith T. K., Carmona-Gutierrez D. (2014). Spermidine feeding decreases age-related locomotor activity loss and induces changes in lipid composition. *PLoS One*.

[132] Grootaert M. O. J., Kurdi A., De Munck D. G., Martinet W., De Meyer G. R. Y., Hayat M. A. (2016). Autophagy in atherosclerosis. *Autophagy: Cancer, Other Pathologies, Inflammation, Immunity, Infection, and Aging*.

[133] Aliwaini S., Bleloch J., Kimani S., Prince S., Hayat M. A. (2016). Induction of autophagy and apoptosis in melanoma treated with palladacycle complexes. *Autophagy: Cancer, Other Pathologies, Inflammation, Immunity, Infection, and Aging*.

[134] Abdelaziz H. M., Freag M. S., Elzoghby A. O., Kesharwani P. (2019). Solid lipid nanoparticle-based drug delivery for lung cancer. *Nanotechnology-Based Targeted Drug Delivery Systems for Lung Cancer*.

[135] Mughal M. H. (2019). Linalool: a mechanistic treatise. *Journal of Nutrition, Food Research and Technology*.

[136] Silva R. O., Sousa F. B., Damasceno S. R. (2014). Phytol, a diterpene alcohol, inhibits the inflammatory response by reducing cytokine production and oxidative stress. *Fundamental & Clinical Pharmacology*.

[137] Santos C. C., Salvadori M. S., Mota V. G. (2013). Antinociceptive and antioxidant activities of phytol *in vivo* and *in vitro* models. *Journal of Neuroscience*.

[138] Alencar M., Islam M. T., Ali E. S. (2018). Association of phytol with toxic and cytotoxic activities in an antitumoral perspective: a meta-analysis and systemic review. *Anti-Cancer Agents in Medicinal Chemistry*.

[139] Islam M. T., Ali E. S., Uddin S. J. (2018). Phytol: a review of biomedical activities. *Food and Chemical Toxicology*.

[140] Islam M. T., de Alencar M. V., da Conceicao Machado K. (2015). Phytol in a pharma-medico-stance. *Chemico-Biological Interactions*.

[141] Islam M. T. (2019). Antipyretic effect of phytol, possibly via 5KIR-dependent COX-2 inhibition pathway. *Inflammopharmacology*.

[142] Islam M. T., Molla S., Das A. K., Zaman F., Khan R. (2019). *In vitro* anti-atherothrombosis activity of *Nigella sativa* oil, phytol and their combinations. *Indian Journal of Pharmaceutical Education and Research*.

[143] Blair M. (2016). Diabetes mellitus review. *Urologic Nursing*.

[144] Hashmi W. J., Ismail H., Mehmood F., Mirza B. (2018). Neuroprotective, antidiabetic and antioxidant effect of *Hedera nepalensis* and lupeol against STZ + AlCl3 induced rats model. *Daru*.

[145] Wang J., Huang M., Yang J. (2017). Anti-diabetic activity of stigmasterol from soybean oil by targeting the GLUT4 glucose transporter. *Food & Nutrition Research*.

[146] Kuete V., Omosa L. K., Tala V. R. (2016). Cytotoxicity of plumbagin, rapanone and 12 other naturally occurring quinones from Kenyan flora towards human carcinoma cells. *BMC Pharmacology and Toxicology*.

[147] Le G., Novotny S. A., Mader T. L. (2018). A moderate oestradiol level enhances neutrophil number and activity in muscle after traumatic injury but strength recovery is accelerated. *The Journal of Physiology*.

[148] Antwi A. O., Obiri D. D., Osafo N., Essel L. B., Forkuo A. D., Atobiga C. (2018). Stigmasterol alleviates cutaneous allergic responses in rodents. *BioMed Research International*.

[149] Kangsamaksin T., Chaithongyot S., Wootthichairangsan C., Hanchaina R., Tangshewinsirikul C., Svasti J. (2017). Lupeol and stigmasterol suppress tumor angiogenesis and inhibit cholangiocarcinoma growth in mice via downregulation of tumor necrosis factor-alpha. *PLoS One*.

[150] Adebiyi O. E., Olopade J. O., Olayemi F. O. (2018). Sodium metavanadate induced cognitive decline, behavioral impairments, oxidative stress and down regulation of myelin basic protein in mice hippocampus: ameliorative roles of beta-spinasterol, and stigmasterol. *Brain and Behavior*.

[151] Zhao C. H., Zhao C., Ye H. Q. (2019). Hypolipidemic activity of low-cholesterol ovum oil of *Rana chensinensis* and phytosterol (stigmasterol) in rats. *Journal of Zhejiang University Science B*.

[152] Xu W., Li L., Sun J. (2018). Putrescine delays postovulatory aging of mouse oocytes by upregulating PDK4 expression and improving mitochondrial activity. *Aging*.

[153] El-Halfawy O. M., Valvano M. A. (2014). Putrescine reduces antibiotic-induced oxidative stress as a mechanism of modulation of antibiotic resistance in *Burkholderia cenocepacia*. *Antimicrobial Agents and Chemotherapy*.

[154] Yang Q., Zheng C., Cao J. (2016). Spermidine alleviates experimental autoimmune encephalomyelitis through inducing inhibitory macrophages. *Cell Death & Differentiation*.

[155] Ohashi K., Kageyama M., Shinomiya K. (2017). Spermidine oxidation-mediated degeneration of retinal pigment epithelium in rats. *Oxidative Medicine and Cellular Longevity*.

[156] Eisenberg T., Abdellatif M., Schroeder S. (2016). Cardioprotection and lifespan extension by the natural polyamine spermidine. *Nature Medicine*.

[157] Yang Y., Chen S., Zhang Y. (2017). Induction of autophagy by spermidine is neuroprotective via inhibition of caspase 3-mediated beclin 1 cleavage. *Cell Death & Disease*.

[158] Tabor C. W., Tabor H. (1984). Polyamines. *Annual Review of Biochemistry*.

[159] Rolen S. H., Sorensen P. W., Mattson D., Caprio J. (2003). Polyamines as olfactory stimuli in the goldfish *Carassius auratus*. *The Journal of Experimental Biology*.

[160] Lefevre P. L., Palin M. F., Murphy B. D. Polyamines on the reproductive landscape. *Endocrine Reviews*.

[161] Scott A. M., Zhang Z., Jia L. (2019). Spermine in semen of male sea lamprey acts as a sex pheromone. *PLoS Biology*.

[162] Zhang M., Caragine T., Wang H. (1997). Spermine inhibits proinflammatory cytokine synthesis in human mononuclear cells: a counterregulatory mechanism that restrains the immune response. *Journal of Experimental Medicine*.

[163] Christian B. E., Haque M. E., Spremulli L. L. (2010). The effect of spermine on the initiation of mitochondrial protein synthesis. *Biochemical and Biophysical Research Communications*.

[164] Wei C., Li H., Wang Y. (2016). Exogenous spermine inhibits hypoxia/ischemia-induced myocardial apoptosis via regulation of mitochondrial permeability transition pore and associated pathways. *Experimental Biology and Medicine*.

[165] Jin J., Li H., Zhao G., Jiang S. (2018). Lycopsamine exerts protective effects and improves functional outcome after spinal cord injury in rats by suppressing cell death. *Medical Science Monitor: International Medical Journal of Experimental and Clinical Research*.

[166] Geetha T., Varalakshmi P. (2001). Anti-inflammatory activity of lupeol and lupeol linoleate in rats. *Journal of Ethnopharmacology*.

[167] Prasad S., Kalra N., Shukla Y. (2007). Hepatoprotective effects of lupeol and mango pulp extract of carcinogen induced alteration in Swiss albino mice. *Molecular Nutrition & Food Research*.

[168] Malinowska M., Miroslaw B., Sikora E. (2019). New lupeol esters as active substances in the treatment of skin damage. *PLoS One*.

[169] Jamshidi-Kia F., Lorigooini Z., Amini-Khoei H. (2018). Medicinal plants: past history and future perspective. *Journal of Herbmed Pharmacology*.

[170] Sarker S. D., Nahar L. (2007). *Chemistry for Pharmacy Students: General, Organic, and Natural Product Chemistry*.

[171] Zhang H. (2008). Bioactive natural products: detection, isolation, and structural determination. *Phytomedicine*.

[172] Clark A. M. (1996). Natural products as a resource for new drugs. *Pharmaceutical Research*.

[173] Bentley R. E. (2018). *Medicinal Plants*.

[174] Ross I. A. (1999). *Constituents, Medicinal Plants of the World (Volume 3): Chemical Traditional and Modern Medicinal Uses*.

[175] Pan S.-Y., Zhou S.-F., Gao S.-H. (2013). New perspectives on how to discover drugs from herbal medicines: CAM’s outstanding contribution to modern therapeutics. *Evidence-Based Complementary and Alternative Medicine*.

[176] Chan K., Shaw D., Simmonds M. S. (2012). Good practice in reviewing and publishing studies on herbal medicine, with special emphasis on traditional Chinese medicine and Chinese materia medica. *Journal of Ethnopharmacology*.

[177] Ma J., Rong K., Cheng K. (2012). Research and practice on biodiversity in situ conservation in China: pro-gress and prospect. *Biodiversity Science*.

